# Extending Our Reach

**Published:** 2004-12-15

**Authors:** Lynne S. Wilcox

With this issue, *Preventing Chronic Disease* (*PCD*) begins its second year of publication. The expertise and enthusiasm of our authors, editors, reviewers, and board members enabled *PCD* to provide our readers with a unique view of public health and chronic disease in 2004:

- Peer-reviewed articles of original research, community case studies, literature reviews, and special topics in public health.
- Guest editorials and commentaries by recognized authorities such as Leonard Syme of the University of California, Berkeley; George Hardy, Jr, of the Association of State and Territorial Health Officials; David Katz of Yale University; Adrian Bauman of Sydney University; and Jane Fountain of Harvard University.
- Educational information for professionals on public health law, social marketing, consortia development, and geographic information systems.
- Information for community advocates on community-based participatory research, conference and media campaigns, and community cancer programs.
- Original art and multimedia presentations related to the populations of public health.

Since we published our first issue, *PCD* has received more than 9000 subscriptions and more than 1.9 million Web site hits. Additionally, the journal has been accepted for indexing in PubMed/Medline, the Directory of Open Access Journals (DOAJ), and the Cumulative Index to Nursing and Allied Health Literature (CINAHL) and will provide all articles to the PubMed Central open access catalog. But beyond these numbers, *PCD* demonstrated that it is possible to create an online journal that achieves its original goals: to establish dialogue between researchers and practitioners; to explore new concepts in chronic disease prevention; and to emphasize multidisciplinary, multisectorial approaches to public health.

We begin our second year by extending *PCD*'s multimedia capacity and offering selected articles in Spanish. We thank Dr. Barbara Bowman of the National Center for Chronic Disease Prevention and Health Promotion, Centers for Disease Control and Prevention, for serving as guest editor for this issue, which features articles that address the public health challenges of diabetes along the border of Mexico and the United States. We have provided Spanish and English translations for selected border health articles and editorials as well as for all border health abstracts. We look forward to hearing from our readers on the usefulness of selected translations.

The border region along the Rio Grande (Rio Bravo) has its own culture, distinct from other communities in either country. The food, the festivals, and the politics are all shared by the *fronterizos*. Chronic disease morbidity is also shared across the border. In the communities described in this issue, 20% of the Mexican Americans older than 39 have diabetes. The Arizona *Border Health ¡SI!* program is multisectoral and designed to encourage prevention and control of diabetes (1).

East of the communities of *Border Health ¡SI!,* the international Bridge of the Americas spans the border between El Paso, Texas, and Ciudad Juarez, Chihuahua. Each country has jurisdiction over half of the bridge, and thousands of cars and pedestrians cross daily in both directions. Chrissie Orr, an artist, recognized that the bridge has ambivalent character:

. . . [T]he caged walkway is a hangout for different kinds of people. . . . It is the "between," the "never never land"; it is the State of Suspended Animation. While working on this project [described below] a baby was born on the bridge. It was born to Mexican parents just past the official border marker on the American side, making the baby girl a full-fledged American citizen. . . . [P]eople line up at the middle of the bridge and sleep here; in the winter they light small fires. . . . (2)

Orr worked with children on both sides of the bridge to create painted shirts that, when hung in the pedestrian walkway, reached across the entire bridge. This year we extend *PCD*'s reach to you, our readers. Thank you for your support in establishing this journal.
